# Moses Judah Folkman 1933–2008

**Published:** 2008-01

**Authors:** P. Gold

On January 14, a bright light in the field of cancer research was extinguished with the sudden death of Dr. Judah Folkman. Folkman first introduced the concept of tumour angiogenesis as a key factor in tumour growth and a target for tumour therapy in 1970. Despite the great skepticism accorded his work, he persisted in his studies. His efforts were eventually vindicated and reproduced in numerous laboratories. Indeed, an enormous number of labs continue to work in the field that Judah Folkman initiated, and about a dozen anti-angiogenic drugs are already on the market.

A native of Cleveland, Ohio, Judah Folkman earned his Bachelor of Arts *cum laude* from Ohio State University in 1953, and his md *magna cum laude* from Harvard Medical School in 1957. While an undergraduate, he worked in Dr. Robert Zollinger’s surgical laboratory and co-authored papers describing a new method of hepatectomy for liver cancer. As a student at Harvard Medical School, he worked in Dr. Robert Gross’s laboratory, where he developed the first atrioventricular implantable pacemaker, for which he received the Boylston Medical Prize, the Soma Weiss Award, and the Borden Undergraduate Award in Medicine.

In 1957, Folkman began his surgical training at the Massachusetts General Hospital, serving as its chief resident in surgery in 1964–1965. That residency was interrupted between 1960 and 1962, when he served as a lieutenant in the U.S. Navy at the National Naval Medical Center in Bethesda. It was there that he, along with colleague Dr. David Long, first demonstrated the sustained release of drugs from implantable silicone rubber polymers that led to the development of the Norplant 5-year contraception system and initiated the field of controlled-release technology. It was also in Bethesda that Folkman carried out experiments growing tumours in isolated perfused organs, which led to the idea that tumours are angiogenesis-dependent, requiring new blood vessels to survive.

In 1965, Folkman joined Harvard’s surgical service, and in 1967, he was promoted to Professor of Surgery at Harvard Medical School and to surgeon-in-chief at the Children’s Hospital Medical Center, becoming the Julia Dyckman Andrus Professor of Pediatric Surgery in 1968.

Folkman’s discoveries related to the mechanism of angiogenesis opened a field of investigation now pursued worldwide. This work elaborated the hypothesis that tumours can grow only if accompanied by the development of feeder blood vessels—that is, angiogenesis—and that malignancy may depend in part on proteins that stimulate vascular growth. His laboratory reported the first purified angiogenesis molecule and the first angiogenesis inhibitor, proposed the concept of angiogenic disease, and initiated clinical trials based on that research. He developed almost all of the methodology for the field, including the first cloning and culture of capillary endothelial cells, and sustained-release polymer technology, together with the *in vivo* bioassays for the various phenomena involved. He elucidated the sequential steps of capillary networks, and he proposed a linkage between cell shape and growth, currently understood in terms of mechanochemical mediation of gene expression by cytoskeletal elements.

Folkman’s exceptional achievements have been recognized with numerous national and international awards. On three separate occasions, he delivered the commencement address at Harvard Medical School. He was elected to the National Academy of Sciences and was a member of the American Academy of Arts and Sciences, the American Philosophical Society, and the Institute of Medicine. Folkman also received a Gairdner Foundation International Award and a Christopher Columbus Discovery Award in Biomedical Research from the National Institutes of Health. He was a recipient of the Wolf Prize in Medicine from Israel. In 1993, he received the Lucian Award and a dsc (Honoris Causa) from McGill University (among his dozen or more honorary degrees).

Judah Folkman was a superb doctor, a brilliant investigator, and a good friend. He will be much missed.

